# Conversion of human glial cells into neurons in *ex vivo* culture of human brain tissue: Essential roles of the transcription factors NeuroD1 and Ascl1

**DOI:** 10.4103/NRR.NRR-D-24-00883

**Published:** 2024-12-07

**Authors:** Liang Xu, Qingsong Wang, Jiancheng Liao, Jiajun Zheng, Bing Qin, Wen Li, Jiaxuan Zhang, Wei Li, Xiangyu Wang, Maoying Zhang, Gong Chen

**Affiliations:** 1 Guangdong-Hongkong-Macau CNS Regeneration Institute of Jinan University, Key Laboratory of CNS Regeneration (Jinan University)-Ministry of Education, Guangdong Key Laboratory of Non-human Primate Research, Guangzhou, Guangdong Province, China; 2 Department of Neurosurgery, The First Affiliated Hospital of Jinan University, Guangzhou, Guangdong Province, China; 3 Department of Anesthesiology, Guangzhou First People’s Hospital, Guangzhou, Guangdong Province, China; 4 Department of Neurosurgery, The Sixth Aﬃliated Hospital of Jinan University, Dongguan, Guangdong Province, China

**Keywords:** Ascl1, brain slice, cell conversion, *ex vivo* culture, glial cell, glia-to-neuron conversion, human brain, *in vivo* reprogramming, neural regeneration, NeuroD1

## Abstract

Transcription factor–mediated cell conversion has been reported in the central nervous system of both rodents and nonhuman primates. In particular, glia-to-neuron conversion has been achieved in the brain and spinal cord of animal models for neural regeneration and repair. However, whether glia-to-neuron conversion can be used for brain repair in humans needs to be explored. To investigate the use of glia-to-neuron conversion technology in the human brain, we established a long-term *ex vivo* culture system using human brain tissue that was surgically removed from epileptic patients to test glia-to-neuron conversion directly. We found that neural transcription factors NeuroD1 and Ascl1 both converted human glial cells into neurons. Immunostaining and electrophysiological recordings showed that the glia-converted neurons demonstrated immature properties during the initial 7–14 days of conversion, and then acquired more mature neuronal properties after 21–27 days of conversion. These *ex vivo* conversion studies in human brain tissue pave the way toward future clinical trials using a transcription factor–based glia-to-neuron conversion approach to treat neurological disorders.

## Graphical abstract

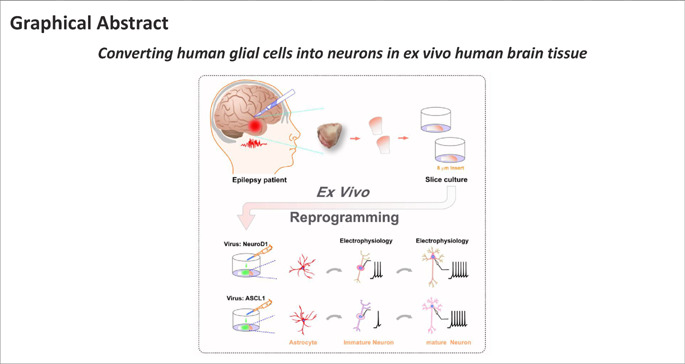

## Introduction

The ability for neural regeneration in the adult mammalian brain and spinal cord is rather limited, except for a few neurogenic niches (Boldrini et al., 2018; Kempermann et al., 2018; Sorrells et al., 2018). In contrast to the limited number of adult neural stem cells, glial cells are widely distributed in the central nervous system and can be activated to divide after brain injury (Pekny and Pekna, 2014; Li et al., 2022). Recently, *in vivo* direct reprogramming through the overexpression of master transcription factors in reactive glial cells has been developed as an approach to generate new neurons in the central nervous system (Guo et al., 2014; Li and Chen, 2016; Bocchi et al., 2022; Liang et al., 2024). In particular, *in vivo* glia-to-neuron (GtN) conversion has been reported to promote functional recovery in rodent models of neurological disorders (Chen et al., 2020; Puls et al., 2020; Wu et al., 2020; Jiang et al., 2021; Zheng et al., 2022). Excitingly, NeuroD1-mediated GtN conversion has been achieved in an ischemic injury model in nonhuman primates (NHPs) (Ge et al., 2020). These studies in both rodents and NHPs indicate that *in vivo* GtN conversion may have potential as a therapeutic approach for neural repair. However, whether such glia conversion therapy would work in humans requires further investigation.

Current preclinical experiments have mainly used rodent models. Because of the structural and functional differences between rodents and humans, many drugs that show promising results in rodents often fail in human clinical trials (Turner et al., 2013; Drummond and Wisniewski, 2017). Recently, the US Food and Drug Administration reduced the requirements for animal studies in new drug development (Wadman, 2023). New strategies to validate drug efficacy is an urgent issue. Human tissue-derived organotypic culture has a three-dimensional structure composed of a variety of cells, retaining certain *in vivo* morphological and physiological characteristics (Le Duigou et al., 2018). However, it is very challenging to culture adult human brain tissue *in vitro* because neurons often die quickly once surgically removed from the brain (Schwarz et al., 2019). Recently, a research team reported the culture of human brain slices with neural activity for up to 6–7 weeks (Le Duigou et al., 2018). Such long-term *ex vivo* culture of human brain slices may provide an important means for studying brain disorders and screening therapeutic drugs (Schommer et al., 2017; Qi et al., 2019).

In this study, we established a long-term *ex vivo* method to culture human brain slices for 1 month and demonstrated that neural transcription factors NeuroD1 and Ascl1 both converted human glial cells into neurons. Moreover, these human glia-converted neurons exhibited electrophysiological properties, including repetitive action potentials and synaptic currents. Our studies provide direct evidence in *ex vivo* human brain tissue that neural transcription factor–mediated GtN conversion may offer a unique strategy for neural regeneration and repair, paving the way toward future human clinical trials.

## Methods

### Animals

Male Sprague–Dawley rats (6–8 weeks old, 200–300 g) were purchased from the Guangdong Medical Laboratory Animal Center, China (license No. SCXK (Jing) 2016-0006), which operates under a barrier system of clean grade. Only male rats were used in this study because female rats’ estrogen can affect the occurrence of epileptic seizures (Veliskova and Desantis, 2013; Zheng et al., 2022). The animals were housed under a 12/12-hour light/dark cycle with controlled temperature (20–26°C) and had access to sufficient food and water. The Animal Experiment Protocol was approved by the Laboratory Animal Ethics Committee of Jinan University (IACUC # 20181211-07) on September 11, 2022 and conducted in strict accordance with the National Institutes of Health Guide for the Care and Use of Laboratory Animals (8^th^ ed., National Research Council, 2011).

### Brain tissue from epilepsy patients

Human brain tissue specimens were obtained after surgery from 11 patients with drug-resistant epilepsy at The First Affiliated Hospital of Jinan University, Guangzhou, China. The basic information of the patients is shown in **Table 1**. The use of resected tissue was approved by the Institutional Review Board at Jinan University (IRB # 2018-024) on May 3, 2018. Informed consent for participating in the study and publication was obtained from each participant. The study was conducted in accordance with the *Declaration of Helsinki*.

**Table 1:** Tissue samples included in this study

Patient	Sex	Age at surgery (yr)	Brain region
1	Male	2	Temporal lobe
2	Male	3	Temporal lobe
3	Male	15	Frontal lobe
4	Male	44	Temporal lobe
5	Male	47	Temporal lobe
6	Male	27	Temporal lobe
7	Female	1	Temporal lobe
8	Female	57	Temporal lobe
9	Female	46	Frontal lobe
10	Female	45	Temporal lobe
11	Male	25	Temporal lobe

### Organotypic slice culture preparation

A sterile working environment was maintained to prevent sample contamination. All tools used during the procedure were sterilized. Brain tissue specimens removed from patients were immediately incubated in an icy cutting solution (80 mM NaCl, 75 mM sucrose, 2.5 mM KCl, 25 mM glucose, 1.25 mM NaH_2_PO_4_, 24 mM NaHCO_3_, 4 mM MgCl_2_, 0.5 mM CaCl_2_, 10 mM N-2-hydroxyethylpiperazine-N-2-ethane sulfonic acid [HEPES], 1% antibiotic-antimycotic, bubbled with 95% O_2_:5% CO_2_, pH 7.3 and osmolarity 300–310 mOsm) (Lee et al., 2018). After removing blood vessels and pia mater, the specimens were cut into 300-μm-thick sections using a vibrating microtome (VT1200; Leica Biosystems, Nussloch, Germany). For rat brain slice culture, rats were anesthetized with 3% isoflurane (RWD, Shenzhen, China) by R500 Small Animal Anesthesia Machine (RWD), and their brains were removed and sectioned into 300-μm-thick slices.

After cutting, brain slices were initially incubated for 30 minutes in Hanks Balanced Salt Solution (HBSS) with HEPES (20 mM; room temperature, bubbled with 95% O_2_:5% CO_2_, pH 7.3, osmolarity 300–320 mOsm). Then, they were incubated successively in HBSS, 50/50 HBSS/brain slice medium (BSM), and BSM (Neurobasal, DMEM/F12, 15% horse serum, 2 mM L-glutamax, 1.7 μM insulin, 5 mM D-glucose, 1% B27, 1% antibiotic mix, 1% N_2_, 1 mM Na pyruvate, 4 mM Na lactate, 1 mM creatine, 200 μM creatine phosphate, 1 μM dorsomorphin, 0.015 mM ATP, 0.5 μM thiazovivin, 10 μM forskolin, 0.58 mM ascorbic acid, 20 µM α-tocopherol, 10 ng/mL platelet-derived growth factor-AA, 10 ng/mL insulin-like growth factor I. Each condition was maintained for 5 minutes. Inserts with 0.4 μm (PICM0RG50 and PTHT06H48, Millipore, Darmstadt, Germany) and 8.0 μm (PTEP06H48, Millipore) pore size were individually placed in the 6-well plate containing 2 mL BSM per well and prewarmed in a 37°C 5% CO_2_ incubator. After the gradual switch from HBSS to BSM, brain slices were placed on membranes of the prewarmed and wet inserts and incubated in a 37°C 5% CO_2_ incubator. The medium was refreshed every day for the first 3 days and then every 2 days afterwards.

### Brain slice whole-cell recordings

The brain slice was placed into a chamber perfused with a recording solution containing (mM): NaCl, 126; KCl, 2.5; glucose, 10; NaH_2_PO_4_, 1.25; NaHCO_3_, 26; MgCl_2_, 2; CaCl_2_, 2, equilibrated with 95% O_2_/5% CO_2_, pH 7.3 and osmolarity 300–310 mOsm, at 26°C. The recording was made in whole-cell mode with a pipette resistance of 4–7 mΩ. The pipette solution contained (mM): K-Gluconate, 126; KCl, 4; HEPES, 10; Mg_2_ATP, 4; Na_2_GTP, 0.3; phosphocreatine, 10 (pH 7.3 adjusted with KOH, and osmolarity 290–300 mOsm). The holding potential for voltage-clamp experiments was –70 mV. Signals were amplified with a Multiclamp 700B amplifier (Molecular Devices, Palo Alto, CA, USA), digitized at 10 kHz, filtered at 1 kHz, and recorded with pClamp 10 and Clampex 10.4 software (Molecular Devices). The data were analyzed with Clampfit 10.4 (Molecular Devices; Zheng et al., 2022).

### Virus production and infection

#### Retroviruses

Because human *NEUROD1* and *ASCL1* genes are quite conserved compared with mouse *Neurod1* and *Ascl1* genes according to BLAST, the sequences of mouse *Ascl1* and *Neurod1* were inserted into a pCAG-GFP-IRES-GFP retroviral vector to generate pCAG-Ascl1-IRES-GFP and pCAG-Neurod1-IRES-GFP vectors. Viral particles were packaged in human embryonic kidney (HEK) 293T cells, currently the most commonly used cell line for retrovirus packaging, to generate vesicular stomatitis virus glycoprotein-pseudotyped retroviruses encoding neurogenic factors in biological safety cabinets (HFsafe 1800LCB2, Heal Force, Shanghai, China). The titer of viral particles was approximately 10^7^–10^8^ particles/mL, determined after transduction of HEK 293T cells. Approximately 3 μL retrovirus was added onto each brain slice for 2 days.

#### Adeno-associated virus

Adeno-associated virus (AAV)9 GFAP::Cre, AAV9 CAG::Flex-GFP and AAV9 CAG::Flex-*Neurod1*-GFP were produced by PackGene Biotech (Guangzhou, China). For the virus concentrations, AAV9 GFAP::Cre was adjusted to 1 × 10^11^ GC/mL, and Flex-GFP and Flex-Neurod1-GFP were adjusted to 1 × 10^12^ GC/mL. The ratio of Cre and Flex was 1:10. The total volume added was 3 μL.

### Immunofluorescence staining

Brain slices were fixed for 12–24 hours in 4% paraformaldehyde at 4°C, and then transferred to 30% sucrose at 4°C overnight. After embedding in Optimal Cutting Temperature (Tissue-Tek®, Sakura® Finetek, Torrance, CA, USA), brain slices were serially sectioned on the CryoStar NX50 (Thermo Fisher Scientific, Walldorf, Germany) at a thickness of 40 μm. For immunostaining, brain slices were washed with phosphate buffer saline (PBS) and blocked for 1 hour at room temperature in blocking solution (10% normal donkey serum, 5% bovine serum albumin, and 0.5% Triton X-100, prepared in PBS), and then incubated overnight at 4°C with primary antibodies diluted in the blocking solution. After three washes with 0.2% tween-20 in PBS, the slices were incubated with secondary antibodies supplemented with 0.5 mg/mL DAPI (F. Hoffmann-La Roche, Natley, NJ, USA) in PBS for 2 hours at room temperature and then washed with PBS. Finally, samples were mounted with VECTASHIELD® mounting medium (VECTOR Laboratories, Burlingame, CA, USA) and sealed with nail polish. Representative images were taken with Zeiss Axioplan fluorescent microscope (Axio Imager Z2, Zeiss, Jena, Germany) or confocal microscope (LSM880, Zeiss).

Primary antibodies used were as follows: mouse anti-glial fibrillary acidic protein (GFAP; 1:1000, Cat# G3893, sigma, St. Louis, MO, USA), rabbit anti-GFAP (1:500, Cat# G9269, Sigma), rabbit anti-neuronal nuclei antigen (NeuN; 1:1000, Cat# ab177487, Abcam, Cambridge, MA, USA), rabbit anti-S100β (1:500, Cat# ab52642, Abcam), chicken anti-microtubule-associated protein 2 (MAP2; 1:1000, Cat# ab5392, Abcam), rabbit anti-doublecortin (DCX; 1:500, Cat# Ab18723, Abcam), mouse anti-synaptic vesicle glycoprotein 2 (SV2; 1:1000, Cat# AB_2315387, DSHB, Iowa City, USA), mouse anti-tubulin β-3 (Tuj1; 1:1000, Cat# T8660, Abcam), mouse anti-NeuroD1 (1:500, Cat# ab60704, Abcam), rabbit anti-glutamate decarboxylase 67 (GAD67; 1:500, Cat# 198013, SYSY), rabbit anti-gamma-aminobutyric acid (GABA; 1:500, Cat# A2052, Sigma), chicken anti-green fluorescent protein (GFP; 1:1000, Cat# ab13970, Abcam), mouse anti-neurofilament 200 (NF-200; 1:500, Cat# N0142, Sigma), rabbit anti-Ascl1 (1:500, Cat# 211327, Abcam), anti-biotin594 (1:1000, Cat#A12922, Thermo Fisher Scientific). Secondary antibodies were: Alexa Fluor® 488-donkey anti-chicken 488 (Cat# 703-545-155, Jackson ImmunoResearch Labs, West Grove, PA, USA), and Alexa Fluor^TM^ 647-donkey anti-rabbit 647 (Cat# A-31573), Alexa Fluor^TM^ Plus 594-goat anti-mouse 594 (Cat# A-32744), Alexa Fluor^TM^ Plus 555-donkey anti-rabbit Alexa Fluor 555 (Cat# A31572), Alexa Fluor^TM^ Plus 555-donkey anti-mouse Alexa Fluor 555 (Cat# A31570), and Alexa Fluor^TM^ Plus 647-donkey anti-mouse Alexa Fluor 647 (Cat# A31571), all from Thermo Fisher Scientific, and were all used at a dilution of 1:1000.

### Data analysis and statistics

All images were analyzed with Zeiss ZEN 2.3 software (blue edition, Göttingen, Germany). For quantitative analysis, GFP-positive cells were counted on at least three different slices. One-way analysis of variance with Sidak’s multiple comparison test or non-paired Student’s *t*-test was used for statistical analysis using Graphpad Prism 6 (GraphPad Software Inc., San Diego, CA, USA). *P* < 0.05 was set as the significant threshold.

## Results

### Establishing *ex vivo* brain slice culture platform

We have previously reported that the master neural transcription factor NeuroD1 can convert glial cells, including astrocytes, into neurons in both rodents and NHPs (Chen et al., 2020; Ge et al., 2020). To further investigate the potential use of GtN conversion technology in humans, we aimed to establish an *ex vivo* human brain slice culture system using brain tissue surgically removed from epileptic patients.

**Figure 1:**
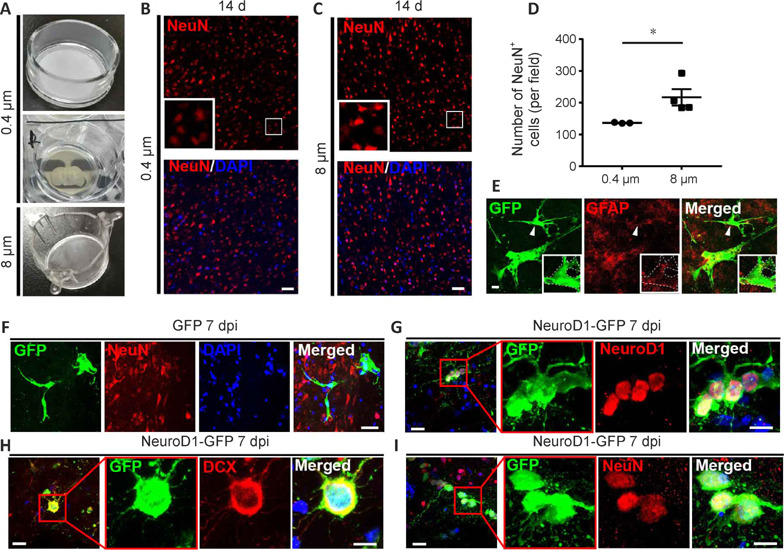
Regenerating neurons in *ex vivo* rat brain slice cultures. (A) Images of cell culture inserts with different diameters: 0.4 μm and 8.0 μm. (B, C) Typical images of neuronal nuclei antigen (NeuN) staining showing neuronal survival at 14 days of rat brain slice culturing in an insert of 0.4 μm (B) or 8 μm (C). (D) Quantification showing more neurons surviving in the 8-μm insert than the 0.4-μm insert after 14 days of culture (*n* ≥ 3). (E, F) Infecting the rat brain slices with control retrovirus expressing green fluorescent protein (GFP) alone revealed glial fibrillary acidic protein (GFAP)-positive astrocytes being infected (E), but no GFP colocalization with NeuN (F) after 7 days post-infection (dpi). (G–I) NeuroD1-GFP-infected cells expressing NeuroD1 (G) were immunopositive for neuronal markers doublecortin (DCX) (H) and NeuN (I). Scale bars: 40 μm for B and C, 10 μm for E–I, and 5 μm for magnified images in G–I. Data are shown as mean ± SEM and statistical significance was determined by unpaired Student’s *t*-test. *P < 0.05.

Initially, we used adult rat brain slices to test *ex vivo* culture conditions. After testing different pore sizes, we selected 8.0 μm hanging inserts for the remainder of our experiments because this insert yielded more surviving neurons after 14 days of culture (**Figure 1A–D**). When we infected *ex vivo* rat brain slices with retroviruses expressing GFP alone (control), we found that GFAP-positive astrocytes were infected (**Figure 1E**), but no NeuN-positive neurons were infected (**Figure 1F**). In contrast, infection with retrovirus Neurod1-GFP resulted in the conversion of glial cells into DCX-positive and NeuN-positive neurons within 7 days (**Figure 1G–I**), consistent with our previous report in the adult mouse brain *in vivo* (Guo et al., 2014).

**Figure 2:**
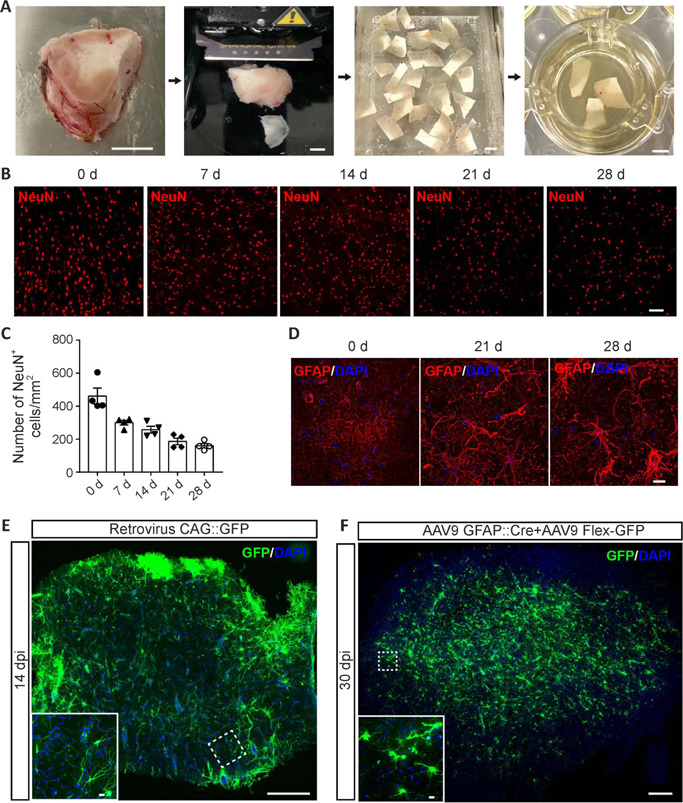
Establishment of *ex vivo* culture of human brain tissue. (A) Illustration of experimental procedures. Human brain tissue was cut into 300-μm slices and incubated in the Hanks Balanced Salt Solution, then transferred to an 8-μm insert for long-term culture. (B) Immunostaining with the neuronal marker neuronal nuclei antigen (NeuN) showed that the surviving neurons gradually decreased from 0 days to 28 days. (C) Quantifications showed the number of surviving neurons decreased (*n* = 4). Data are shown as mean ± SEM. (D) Survival of human astrocytes in the *ex vivo* human brain slices. Astrocytic marker: Glial fibrillary acidic protein (GFAP). (E, F) Human brain slices efficiently infected by retroviruses (E) and adeno-associated virus (AAV) (F). Scale bars: 5 mm for A, 100 μm for B, 20 μm for D, 500 μm for E and F, and 10 μm for magnified images in E and F. dpi: Day(s) post-infection; GFP: green fluorescent protein.

After successfully culturing rat brain slices *ex vivo*, we applied the same strategy to culture human brain slices *ex vivo* using brain tissue after surgical operations (**Figure 2A**). We succeeded in keeping human brain cells alive for at least 1 month, and sometimes even 2 months, after surgical removal. Immunostaining with the neuronal marker NeuN showed that the number of surviving neurons gradually decreased in *ex vivo* culture, with 35% of neurons still surviving after 1 month (**Figure 2B** and **C**). Astrocytes also survived for at least 1 month, as demonstrated by GFAP staining (**Figure 2D**), making it possible to test whether these human astrocytes can be converted into neurons by neural transcription factors.

Next, we tested which types of viruses might be suitable for infecting human brain slices and found that both retroviruses and AAV showed successful infection (**Figure 2E** and **F**), establishing a method for viral delivery of transcription factors to human brain slices in long-term cultures.

### NeuroD1 converts human glial cells into neurons

**Figure 3:**
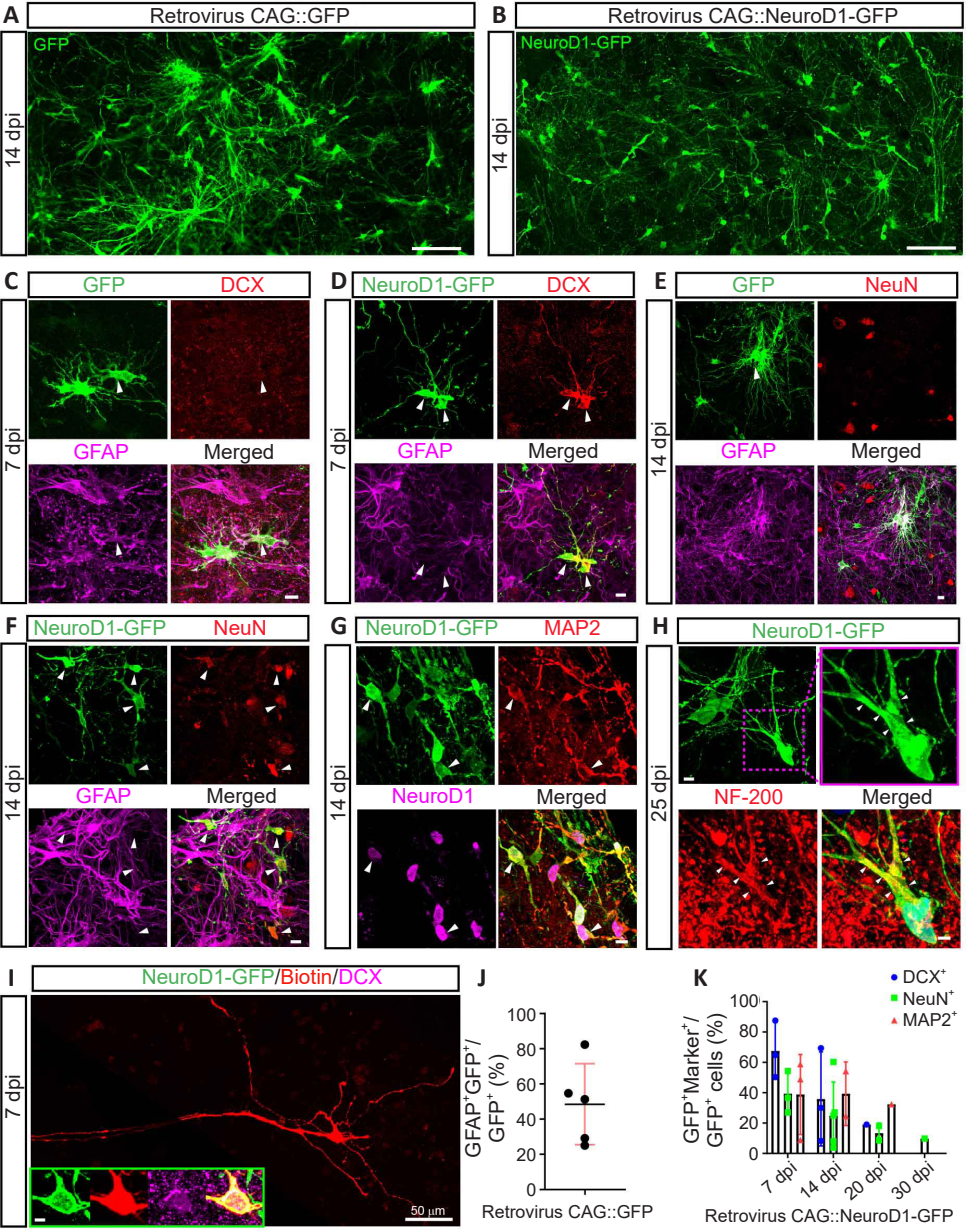
NeuroD1 converts human glia into neurons in human brain slices. (A, B) Typical images showing human brain cells infected by control (A) and NeuroD1 (B) retroviruses. Note the distinct morphology between the control cells and NeuroD1-infected cells. (C, D) At 7 dpi, control virus green fluorescent protein (GFP)-infected cells were partially glial fibrillary acidic protein (GFAP, astrocyte marker)-positive and doublecortin (DCX, immature neuronal marker)-negative (C); whereas, NeuroD1-GFP-infected cells were DCX-positive and GFAP-negative (D). (E, F) At 14 dpi, control virus GFP-infected cells were also GFAP-positive and NeuN-negative (E), whereas NeuroD1-GFP-infected cells were NeuN-positive and GFAP-negative (F). (G, H) NeuroD1-GFP-infected cells were also immunopositive for neuronal markers microtubule-associated protein 2 (MAP2) (G) and neurofilament 200 (NF-200) (H). (I) Biotin was included in the pipette solution during electrophysiological recording (see Figure 6) of the NeuroD1-GFP-converted neurons. Immunostaining of biotin revealed complex morphology of the NeuroD1-GFP-converted neurons. A long process was observed at 7 dpi that was immunopositive for DCX. (J) Quantifications showed that approximately 48.5% of control virus GFP-infected cells were GFAP-positive astrocytes (*n* = 5). (K) Quantifications showed different conversion efficiency at different time points after NeuroD1-GFP infection (*n* = 6). Scale bars: 100 μm for A and B, 10 μm for C–H, 50 μm for I, and 5 μm for insert in I (higher magnification).

We first investigated whether NeuroD1 converts human glial cells into neurons in *ex vivo* human brain tissue culture. To avoid potential neuronal leakage, we first selected retroviruses to infect dividing glial cells only, without infecting non-dividing neurons. Control retroviruses expressing GFP alone infected human astrocytes in the slice culture (**Figure 3A**), as shown by coimmunostaining with the astrocytic marker GFAP (**Figure 3C** and **E**; quantified in **Figure 3J**, where 48.5% of GFP-positive cells were GFAP-positive, *n* = 5 samples). As expected, we did not find any GFP-positive neurons because they do not divide and typically cannot be infected by retroviruses. Because of the limited availability of human brain slices, this study focused on astrocytes rather than microglia or NG cells. This is because during our previous study (Guo et al., 2014) in mammalian brains, our NeuroD1 AAV mainly infected astrocytes and neurons *in vivo*, and was rarely expressed in microglia or NG2 cells.

Glial cells infected by retroviruses expressing NeuroD1-GFP showed dramatic morphological changes toward neuron-like cells with unipolar or bipolar long processes (**Figure 3B**). These cells were immunopositive for a variety of neuronal markers, specifically DCX (**Figure 3D**), NeuN (**Figure 3F**), MAP2 (**Figure 3G**), and NF-200 (**Figure 3H** and **K**). Time course studies showed that NeuroD1-GFP-infected cells were also positive for the immature neuronal marker DCX during the early conversion stage (**Figure 3D**, 7 days postinfection [dpi]) and, at the later stage (14–25 dpi), were positive for the more mature neuronal markers MAP2, NeuN, and NF-200 (**Figure 3F–H**). **Figure 3K** shows the percentages of NeuroD1-GFP-infected cells positive for neuronal markers from 7 to 30 dpi. Overall, the conversion efficiency was 40%–60% at 7 dpi, and then dropped to approximately 20% at 20 dpi (**Figure 3K**), indicating that some newly converted immature neurons might not survive long-term in the human brain slice culture condition. NeuroD1 signal was also clearly detected in the converted neurons (**Figure 3G**). Moreover, during electrophysiological recordings of glia-converted neurons in human brain slices, the NeuroD1-GFP-converted cells were labeled with biotin. Post-recording immunostaining revealed a complex neuron-like morphology of the NeuroD1-GFP-converted cells with long processes at 7 dpi, which were immunopositive for DCX (**Figure 3I**). Together, the results suggested that NeuroD1 converted human glial cells, including astrocytes, into neurons in *ex vivo* human brain tissue.

### Ascl1 converts human glial cells into neurons

**Figure 4:**
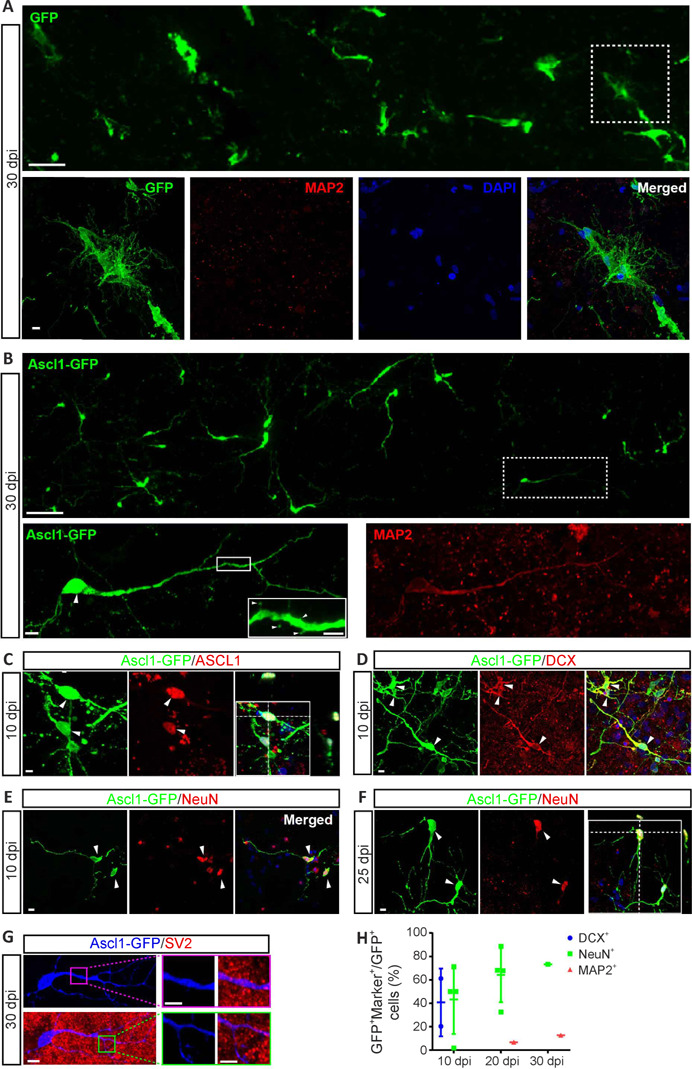
Ascl1 converts human glial cells into neurons in human brain slices. (A) Control virus green fluorescent protein (GFP)-infected cells had glial morphology and did not express microtubule-associated protein 2 (MAP2) at 30 dpi. (B) Ascl1-GFP-infected cells had unipolar or bipolar neuronal morphology and were colocalized with the neuronal marker MAP2 (red). Note that the converted neurons also showed dendritic spines (insert, arrowhead). (C) Confirmation of Ascl1 expression in the converted neurons. (D, E) Ascl1-converted neurons were immunopositive for doublecortin (DCX) or mature neuronal marker neuronal nuclei antigen (NeuN) at 10 dpi. (F) Ascl1-converted neurons colocalized with NeuN and had long neuronal process at 25 dpi. (G) Synaptic puncta (SV2, red) were observed on the neuronal processes of Ascl1-converted neurons (blue, 30 dpi). (H) Quantifications showed different conversion efficiencies at different time points after Ascl1-GFP infection (*n* = 4). Scale bars: 100 μm for A (top row) and B (top row); 10 μm for A (bottom row), B (bottom row), D–G; 5 μm for small insert in B (higher magnification), C, and images in G (higher magnification).

We further investigated whether another pioneer factor, Ascl1, could convert human glial cells into neurons. Cells infected by the control retrovirus expressing GFP alone showed no neuronal marker MAP2 staining (red; **Figure 4A**). Ascl1-GFP-infected cells were observed after 30 days of overexpressing Ascl1 (**Figure 4B**). Ascl1-infected cells displayed typical neuronal morphology with long apical dendrites and were immunostained with neuronal marker MAP2 (**Figure 4B**), indicating that Ascl1 expression dramatically changed the cell morphology. We also confirmed Ascl1 expression in the Ascl1-GFP-infected cells (**Figure 4C**). Time course studies revealed that similar to NeuroD1, Ascl1-GFP-infected cells were initially positive for DCX (**Figure 4D** and **E**, 10 dpi) and later were positive for NeuN (**Figure 4F**, 25 dpi) and synaptic marker SV2 on the dendrites of Ascl1-converted neurons (**Figure 4G**). **Figure 4H** shows the percentages of Ascl1-GFP-infected cells with various neuronal markers. Together, these results suggest that Ascl1 also converted human glial cells into neurons in *ex vivo* human brain tissue.

### Neuronal identity after conversion by NeuroD1 or Ascl1

**Figure 5:**
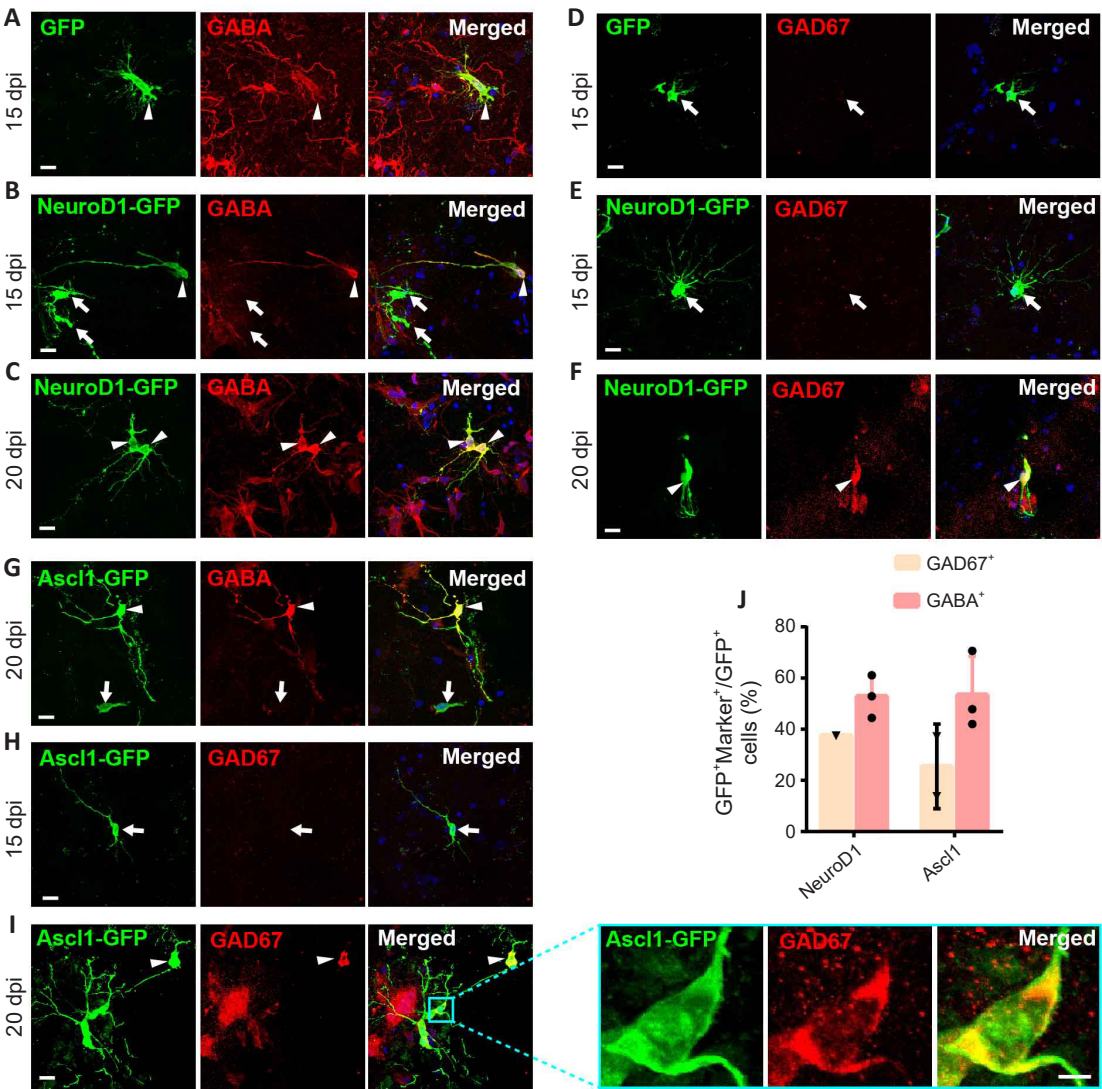
Neuronal identity after conversion by NeuroD1 or Ascl1. (A) Control virus green fluorescent protein (GFP)-infected human astrocytes were immunopositive for gamma-aminobutyric acid (GABA). (B, C) NeuroD1-GFP-infected cells were partially immunopositive for GABA (arrowheads). White arrows point to GABA-negative cells. (D) Control virus GFP-infected cells were immunonegative for the GABAergic marker glutamate decarboxylase 67 (GAD67). (E, F) NeuroD1-GFP-infected cells were GAD67-negative at 15 dpi (E) and GAD67-positive by 20 dpi (F). (G) Ascl1-GFP-infected cells were partially positive for GABA. (H, I) Ascl1-GFP-infected cells were GAD67-negative at 15 dpi (H) and GAD67-positive at 20 dpi (I). (J) Percentages of GABA-positive or GAD67-positive cells among NeuroD1-GFP- or Ascl1-GFP-infected cells (*n* = 3). Scale bars: 20 μm for A–I and 5 μm for higher magnification images in I.

We next examined neuronal identity after conversion by NeuroD1 or Ascl1. Previous studies have reported that human astrocytes are GABA-positive in pathological conditions (Lee et al., 2011; Jo et al., 2014). We therefore performed GABA immunostaining and confirmed that many human astrocytes were indeed GABA-positive (**Figure 5A**). Additionally, many NeuroD1-converted neurons and Ascl1-converted neurons were GABA-positive (**Figure 5B**, **C**, and **G**). To further investigate whether these converted neurons were indeed GABAergic neurons, we performed additional immunostaining with another GABAergic neuron marker GAD67, an enzyme that produces GABA. We found that NeuroD1-converted neurons were initially GAD67-negative at 15 dpi (**Figure 5E**), but later were GAD67-positive at 20 dpi (**Figure 5F**). Control virus GFP-infected cells were GAD67-negative at 15 dpi (**Figure 5D**). Similarly, Ascl1-converted neurons were initially GAD67-negative at 15 dpi (**Figure 5H**), but then became GAD67-positive at 20 dpi (**Figure 5I**). Quantitatively, more than 50% of NeuroD1-converted neurons showed a GABA-positive signal, and less than 40% of NeuroD1-converted neurons showed a GAD67-positive signal (**Figure 5J**). Similarly, more than 50% of Ascl1-converted neurons were GABA-positive, whereas less than 30% of Ascl1-converted neurons were GAD67-positive (**Figure 5J**). We also performed immunostaining using the glutamatergic neuron marker vGlut1, but did not detect any signal. It was unclear whether this was due to a problem with the antibodies we used or a problem with the human slice culture samples.

**Figure 6:**
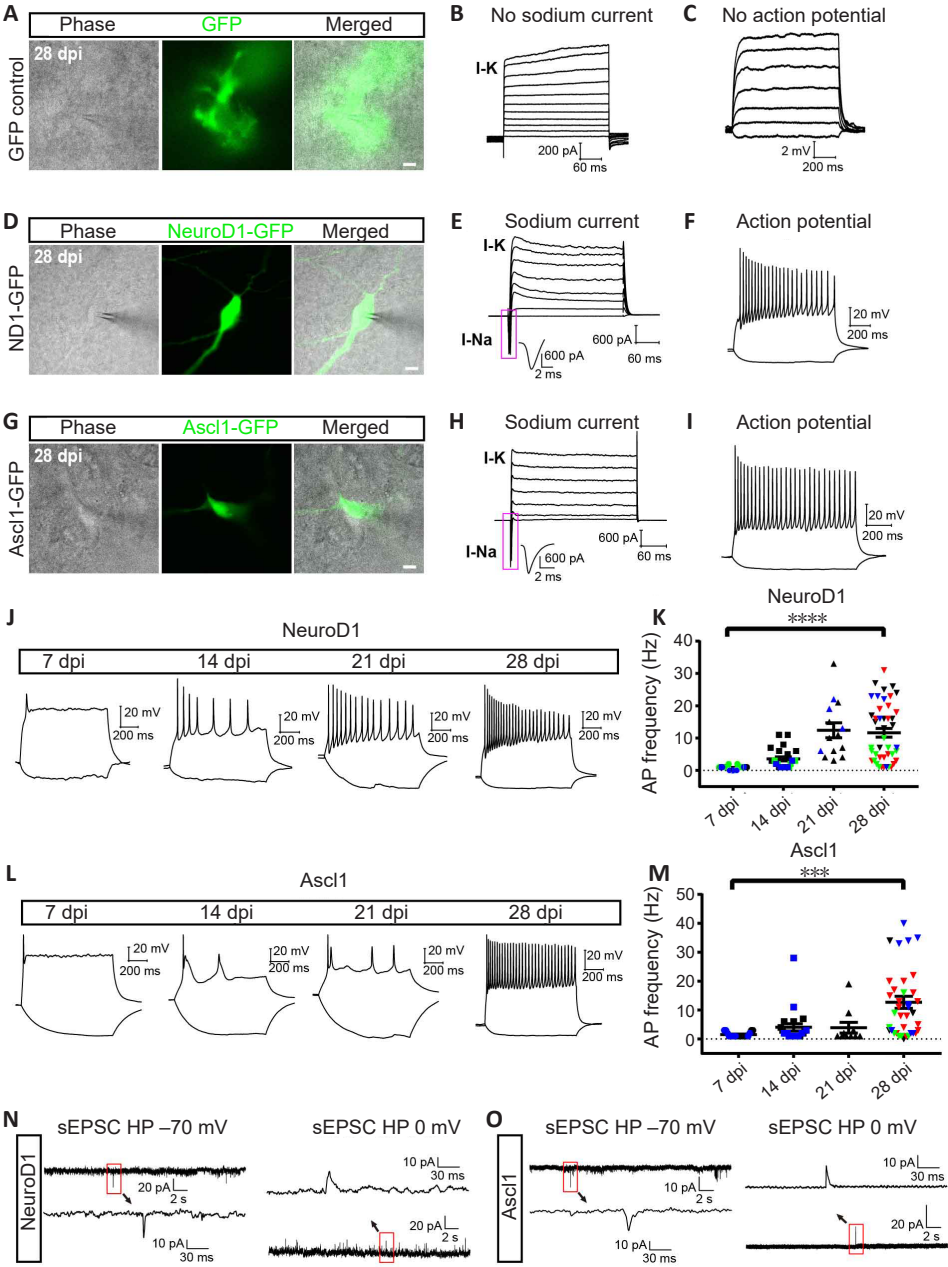
Functional characterization of NeuroD1- and Ascl1-converted neurons in human brain slice recordings. (A) Phase and fluorescent images of control virus green fluorescent protein (GFP)-infected cells. (B, C) Control virus GFP-infected cells had K^+^ currents and lacked Na^+^ currents (B), and displayed no action potentials (C). (D) Typical images of NeuroD1-GFP infected cells. (E, F) NeuroD1-GFP-infected cells showed both K^+^ and Na^+^ currents (E) and repetitive action potentials (F). Scale bars: 10 μm. (G) Representative images of Ascl1-GFP-infected cells. (H, I) Ascl1-GFP-infected cells also showed both K^+^ and Na^+^ currents (H) and repetitive action potentials at 28 dpi (I). (J) Representative traces showing the action potential firing pattern at different time points after NeuroD1-GFP infection. NeuroD1-converted neurons showed repetitive action potentials at 14 dpi. (K) Quantification showed a steady increase in the action potential (AP) frequency among NeuroD1-converted neurons (cell number = 104, number of patients = 4). (L) Typical traces showing the action potential firing pattern at different time points after Ascl1-GFP infection. (M) AP frequency change among Ascl1-converted neurons (cell number = 89, number of patients = 4). (N, O) Typical traces of spontaneous excitatory synaptic currents (sEPSCs) and spontaneous inhibitory synaptic currents (sIPSCs) recorded from NeuroD1-converted neurons (N) or Ascl1-converted neurons (O). Data are shown as mean ± SEM, and statistical significance was determined by one-way analysis of variance with Sidak’s multiple comparisons test. ***P < 0.001, ****P < 0.0001.

### Functional properties of the converted neurons

Using whole-cell patch clamp recording, we investigated whether the human glia-converted neurons were functional. As a control, we recorded cells infected by retroviruses expressing GFP alone (**Figure 6A**, 28 dpi). We found that they showed typical astrocytic electrical properties with large potassium currents, no sodium currents (**Figure 6B**) and no action potentials (**Figure 6C**). In contrast, NeuroD1-GFP-infected cells (**Figure 6D**) showed both sodium and potassium currents (**Figure 6E**) and repetitive action potentials (**Figure 6F**). Ascl1-converted neurons (**Figure 6G**) also showed sodium and potassium currents (**Figure 6H**) and repetitive action potentials at 28 dpi (**Figure 6I**). Time course studies showed that NeuroD1-converted neurons functionally matured earlier than those converted by Ascl1. Specifically, NeuroD1-converted neurons showed a single spike at 7 dpi, and from 14 dpi started to display repetitive action potentials (**Figure 6J**). The frequency of action potentials continuously increased at 21 dpi and 28 dpi (**Figure 6J** and **K**). In contrast, Ascl1-converted neurons showed a rather immature action potential pattern at 7–21 dpi, and it was only at 28 dpi that they started to show a more mature pattern of repetitive active potentials (**Figure 6L** and **M**). Importantly, we detected spontaneous synaptic events in NeuroD1- and Ascl1-converted neurons in whole-cell recordings (**Figure 6N** and **O**), suggesting that these converted neurons formed functional synapses with other neurons. Together, these electrophysiological results suggested that NeuroD1- and Ascl1-converted neurons were initially immature during the first 14 days of conversion and gradually became mature neurons at 21–28 days after conversion.

### AAV NeuroD1-mediated cell conversion

**Figure 7:**
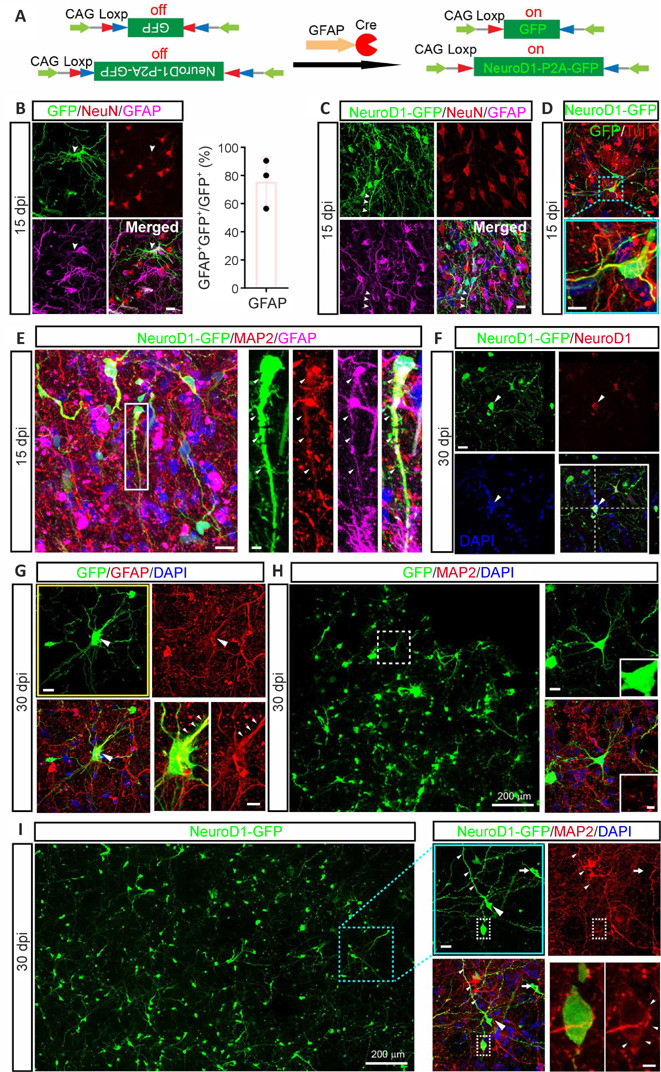
AAV NeuroD1-mediated cell conversion. (A) Schematic diagram illustrating the Cre-FLEX expression system of AAV NeuroD1. GFAP::Cre was used to drive FLEX-NeuroD1 expression in human astrocytes. (B) Control AAV expressing green fluorescent protein (GFP) alone infected GFAP-positive astrocytes as expected, with no NeuN signal (*n* = 3). (C–E) At 15 dpi, AAV NeuroD1-infected cells were partially immunopositive for GFAP (C), indicating their origination from astrocytes, and partially immunopositive for neuronal marker tubulin β-3 (Tuj1) (red, D) and MAP2 (red, E), suggesting partial conversion into neurons. The arrowhead pointing to the GFAP signal in the right enlarged insets. (F) NeuroD1 signal was confirmed in AAV NeuroD1-GFP-infected cells. (G–I) Comparison between AAV GFP-infected cells and AAV NeuroD1-GFP-infected cells at 30 dpi showed remarkable differences in cell morphology. Control AAV GFP-infected cells had a typical astrocytic morphology with star-like branches, and were GFAP-positive (G) and microtubule-associated protein 2 (MAP2)-negative (H), whereas AAV NeuroD1-GFP-infected cells had unipolar or bipolar neuronal morphology and were MAP2-positive at 30 dpi (I). Scale bars: 10 μm for B–E (left), F–H (right); 5 μm for magnified images in D–I; and 200 μm for H (left panel) and I (left panel). AAV: Adeno-associated virus; DAPI: 4′,6-diamidino-2-phenylindole; dpi: day(s) post-infection; GFAP: glial fibrillary acidic protein.

After demonstrating that retroviruses expressing NeuroD1 or Ascl1 converted human glial cells into neurons, we further investigated whether AAV can be used to deliver neural transcription factors for glia conversion in human brain slices. The advantage of AAV is that it can target both dividing and non-dividing astrocytes to increase conversion efficiency. Importantly, AAV has been widely used as a viral vector for gene therapy approved by the US Food and Drug Administration (Naso et al., 2017). To track astrocyte-converted neurons in human brain slices, we developed a Cre-Flex (flip-excision) system that contained a vector expressing Cre recombinase in astrocytes under the control of a GFAP promoter and a Flex vector with an inverted coding sequence of GFP or NeuroD1-P2A-GFP (**Figure 7A**).

We first tested the control AAV expressing GFP alone and found that approximately 75% of the infected cells were GFAP-positive astrocytes, and no expression was found in NeuN-positive neurons (**Figure 7B**), suggesting that our AAV system was rather specific for astrocytes. At 15 dpi, AAV NeuroD1-infected cells had largely lost the GFAP signal (**Figure 7C**) and had acquired the neuronal signals Tuj1 (**Figure 7D**) and MAP2 (**Figure 7E**, note the long apical dendrite). Importantly, some NeuroD1-converted neurons still retained certain astrocytic properties, such as a weak GFAP signal at 15 dpi (**Figure 7E**), suggesting that these cells were in transition from astrocytes to neurons. We also confirmed that cells infected by AAV NeuroD1-GFP expressed the NeuroD1 signal, as expected (**Figure 7F**). By 30 dpi, the control AAV GFP-infected cells remained astrocytes, as shown by GFAP immunostaining (**Figure 7G** and **H**). In contrast, AAV NeuroD1-infected cells mostly displayed unipolar or bipolar neuronal morphology with MAP2-positive signals (**Figure 7I**). Altogether, our results demonstrated that human astrocytes in *ex vivo* brain tissue surgically removed from epileptic patients could be converted into neurons.

## Discussion

In this study, we demonstrated that the neural transcription factors NeuroD1 and Ascl1 both converted human glial cells into neurons in human brain slices surgically removed from epileptic patients. These newly converted neurons initially displayed unipolar or bipolar neuronal morphology and were immunopositive for the immature neuronal marker DCX. After 20–30 days of conversion, the converted neurons were positive for the more mature neuronal markers NeuN and MAP2, and acquired functional properties, namely action potentials and synaptic currents, as demonstrated by whole-cell electrophysiological recordings. AAV-mediated transdifferentiation experiments using the astrocyte-specific promoter GFAP to drive the expression of neural transcription factors further demonstrated that NeuroD1 converted human astrocytes into neurons. The direct demonstration of astrocyte-to-neuron conversion in *ex vivo* human brain slices suggests that such *in vivo* cell conversion technology has potential for development as a therapy for brain repair.

Long-term culture of human brain slices in an *ex vivo* condition is a very challenging task. The most significant challenge is the lack of an energy supply, such as nutrients and oxygen, from blood circulation in the surgically removed brain tissue. Therefore, simulating the living environment of brain tissue in the body is critical for the long-term culture of human brain slices (Le Duigou et al., 2018). Some researchers have reported the use of human cerebrospinal fluid to culture brain tissue to increase cell survival (Schwarz et al., 2017), but obtaining human cerebrospinal fluid in large quantities is difficult. Another challenge for *ex vivo* human brain slice culture is maintaining the three-dimensional structure of the human brain tissue. A previous study has cultured brain slices on semi-permeable membranes with a pore size of 0.4 μm (Ravi et al., 2019) to enable the tissue to absorb nutrients in the medium through the pores and absorb oxygen from the incubator environment. In this study, we tested different supporting membranes with different pore sizes on rat brain tissue and found that a semi-permeable membrane with a pore size of 8 μm was more conducive to the survival of the brain slices. This may be because the larger pore size allows for more nutrients to be absorbed by the brain tissue. Long-term culture of human brain tissue may offer a method for broad testing of therapeutic candidates before conducting human clinical trials.

It is worth noting that during our study, we observed significant variation between different human brain slices in terms of neuronal survival and viral infection. One reason might be because the surgical specimens were obtained from patients of different ages. Another factor for heterogeneity may be the precise location of the brain tissue, even though they are all removed from epileptic patients. Future studies are necessary to characterize the regional differences and single-cell profiles of the converted neurons.

We have previously demonstrated that NeuroD1 converts astrocytes into neurons both *in vitro* and *in vivo*, in rodents and NHPs (Guo et al., 2014; Chen et al., 2020; Ge et al., 2020; Zhang et al., 2020; Ma et al., 2022; Wang et al., 2022; Zheng et al., 2022). This study further demonstrated that NeuroD1 converted human astrocytes into neurons in *ex vivo* human brain tissue, supporting the potential use of glial conversion technology in neural regeneration and repair. To avoid potential neuronal leakage problems, this study mainly used retroviruses to express transcriptional factors NeuroD1 and Ascl1 because retroviruses primarily infect dividing cells, such as glial cells, but do not infect non-dividing cells, such as neurons. This confirms that the retroviruses NeuroD1- and Ascl1-converted neurons originated from dividing glial cells, not from previously existing neurons. After demonstrating GtN conversion using retroviruses, we further tested AAV as the delivering vector because AAV has low pathogenicity in humans and is often selected as the first choice for gene therapy vectors (Grieger and Samulski, 2012; Hastie and Samulski, 2015; Naso et al., 2017). We found that similar to retroviruses, overexpression of NeuroD1 through AAV vectors converted human astrocytes into neurons in *ex vivo* human brain slices. Although the same transcription factor NeuroD1 was expressed for conversion, it appeared that retrovirus-mediated glial conversion proceeded faster than that mediated by AAV, which is consistent with our observations in animal models (Guo et al., 2014; Chen et al., 2020). Presumably, retrovirus-infected dividing glial cells are more sensitive to neural transcription factors, whereas AAV-infected astrocytes may not be dividing and may take longer to respond to neural transcription factors.

Both NeuroD1 and Ascl1 are bHLH family transcriptional factors, critical in neurogenesis during embryonic brain development (Oproescu et al., 2021) and neuronal conversion (Bocchi et al., 2022; Zhang et al., 2022). Interestingly, NeuroD1 has also been found in adult neural stem cells in the mouse hippocampus (Gao et al., 2009). Ascl1 has been reported to induce GABAergic genes in dorsal telencephalic progenitors at early (E12.5) embryonic stages (Casarosa et al., 1999). A recent study on Ascl1‐mediated astrocyte conversion found that NeuroD4 was a downstream target (Rao et al., 2021), whereas our RNA‐sequencing results showed that during NeuroD1‐mediated astrocyte-to-neuron conversion, NeuroD6 and NeuroD2, but not NeuroD4, were significantly upregulated (Ma et al., 2022), suggesting that NeuroD1 and Ascl1 may activate distinct downstream effectors to trigger astrocyte-to-neuron conversion (Bocchi et al., 2022). Interestingly, in the present human brain slice culture study, we found that 25%–40% of NeuroD1- and Ascl1-converted neurons were GAD67-positive GABAergic neurons, which is much higher than that in the mouse cortex (approximately 10%) (Guo et al., 2014; Chen et al., 2020). Such a difference may be due to the astrocytic differences between species, because mouse astrocytes are normally immunonegative for GABA, whereas many human astrocytes are GABA-positive. The heterogeneity of astrocytes between different brain regions may also account for such differences. For example, in the rat hippocampus after the induction of epilepsy, NeuroD1 converts rat hippocampal astrocytes into GABAergic neurons more than that in the cortex (Zheng et al., 2022). These studies may have important implications for future clinical applications. One potential implication is that NeuroD1 gene therapy may produce more GABAergic neurons when applied to human patients, which would be advantageous because more GABAergic neurons may reduce the possibility of epilepsy (Zheng et al., 2022).

A significant challenge for the long-term *ex vivo* culture of human brain slices is that many antibodies that work well in rodents often do not work in human brain slice samples. To better characterize the identity of newly converted neurons, we need to develop new technologies, such as single-cell RNA-sequencing, to fully understand the newly generated neuronal properties in human brain slices.

In conclusion, we demonstrated GtN conversion in human brain tissue resected from epileptic patients. The long-term *ex vivo* culture of human brain tissue may provide a way to screen potential therapeutic agents and facilitate the development of new drug targets. Combined with our previous studies of *in vivo* reprogramming in mice (Chen et al., 2020), rats (Zheng et al., 2022), and NHPs (Ge et al., 2020), this study further extends GtN conversion to human glial cells in *ex vivo* human brain tissue, suggesting that GtN conversion is a highly conserved phenomenon across different species of mammals. However, it is essential to exercise caution when extrapolating these results because we used an *ex vivo* culture system, and human astrocytes may have a different transcriptome compared with those *in vivo*. Another limitation of this type of study is the long-term survival of neurons, which remains a significant challenge. So far, we have been able to keep one-third of human brain neurons surviving after 1 month of *ex vivo* culture. Improving survival to maintain two-thirds of human brain neurons for more than 1 month would further broaden the applications of such *ex vivo* human brain slice culture in pathological investigation and drug screening. Alternatively, because accessing human brain tissue is rather limited, future studies may explore the possibility of using human brain organoids to study human astrocyte conversion or transplantation.

## Data Availability

*No additional data are available.*
